# Effects of Chronic Comorbidities on the Health-Related Quality of Life among Older Patients after Falls in Vietnamese Hospitals

**DOI:** 10.3390/ijerph16193623

**Published:** 2019-09-27

**Authors:** Hai Minh Vu, Long Hoang Nguyen, Tung Hoang Tran, Kiet Tuan Huy Pham, Hai Thanh Phan, Hieu Ngoc Nguyen, Bach Xuan Tran, Carl A. Latkin, Cyrus S.H. Ho, Roger C.M. Ho

**Affiliations:** 1 Department of Trauma and Orthopaedic, Thai Binh Medical University Hospital, Thai Binh 410000, Vietnam; vuminhhai.ythaibinh@gmail.com; 2 Center of Excellence in Behavioral Medicine, Nguyen Tat Thanh University, Ho Chi Minh City 700000, Vietnam; longnh.ph@gmail.com (L.H.N.);; 3 Institute of Orthopaedic and Trauma Surgery, Vietnam—Germany Hospital, Hanoi 100000, Vietnam; tranhoangtung.vd@gmail.com; 4 Institute for Preventive Medicine and Public Health, Hanoi Medical University, Hanoi 100000, Vietnam; phamhuytuankiet_vkt@fpt.vn (K.T.H.P.); bach.ipmph@gmail.com (B.X.T.); 5 Institute for Global Health Innovations, Duy Tan University, Da Nang 550000, Vietnam; 6 Centre of Excellence in Artificial Intelligence in Medicine, Nguyen Tat Thanh University, Ho Chi Minh City 700000, Vietnam; ngochieu.coentt@gmail.com; 7 Bloomberg School of Public Health, Johns Hopkins University, Baltimore, MD 21205, USA; carl.latkin@jhu.edu; 8 Department of Psychological Medicine, National University Hospital, Singapore 119074, Singapore; cyrushosh@gmail.com; 9 Department of Psychological Medicine, Yong Loo Lin School of Medicine, National University of Singapore, Singapore 119228, Singapore; 10 Institute for Health Innovation and Technology (iHealthtech), National University of Singapore, Singapore 119077, Singapore

**Keywords:** health-related quality of life, comorbidity, fall, older, Vietnam

## Abstract

Although comorbidities are prevalent in older people experiencing falls, there is a lack of studies examining their influence on health-related quality of life (HRQOL) in this population. This study examines the prevalence of comorbidities and associations between comorbidities and HRQOL in older patients after falls in Vietnamese hospitals. A cross-sectional design was employed among 405 older patients admitted to seven hospitals due to fall injuries in Thai Binh province, Vietnam. The EuroQol-5 Dimensions-5 Levels (EQ-5D-5L) was used to measure HRQOL. Socio-demographic characteristics were collected using a structured questionnaire, while comorbidities and other clinical characteristics were examined by physicians and extracted from medical records. Multivariate Tobit regression was used to determine the associations between comorbidities and HRQOL. Among 405 patients, 75.6% had comorbidities, of which hypertension and osteoarthritis were the most common. Lumbar spine/cervical spine diseases (Coefficient (Coef.) = −0.10; 95%CI = −0.18; 0.03) and stroke (Coef. = −0.36; 95%CI = −0.61; −0.10) were found to be associated with a significantly decreased EQ-5D index. Participants with three comorbidities had EQ-5D indexes 0.20 points lower (Coef. = −0.20; 95%CI = −0.31; −0.09) in comparison with those without comorbidities. This study underlined a significantly high proportion of comorbidities in older patients hospitalized due to fall injuries in Vietnam. In addition, the existence of comorbidities was associated with deteriorating HRQOL. Frequent monitoring and screening comorbidities are critical to determining which individuals are most in need of HRQOL enhancement.

## 1. Introduction

Falls are well-recognized as a leading health problem in the older population [1]. The World Health Organization refers to a fall as “an event which results in a person coming to rest inadvertently on the ground or floor or other lower-level” [1]. It is estimated that more than 33% of community-dwelling older adults suffer from at least one fall each year, and approximately half of these individuals have recurrent falls [2,3]. People who fall are more likely to experience severe injuries or fractures, impaired mobility, hospitalization, and even premature death [4,5]. Additionally, falls reduce autonomy and social activities, elevate dependence and fear of falls, and result in the deterioration of quality of life [3,6,7]. Therefore, given the rapid growth of an aging population in Vietnam, which is only set to increase further in the coming decades, developing effective strategies for fall prevention, treatment, and care in older adults is critical.

Older people who fall, particularly those requiring medical attention such as hospitalization or emergency department (ED) admission, are more likely to have comorbidities than those without falls [8,9,10,11]. Comorbidity is associated with reduced recovery and increased risk of long-term disability and mortality [9,11,12,13,14]. Previous literature indicates that the prevalence of comorbidities in older patients with falls ranges from 25.8% to 84.1% [8,10,15]. Comorbidity raises a great challenge in providing care and treatment for older adults with falls because, along with fall treatment, they require rigorous care planning to control and manage these comorbidities appropriately [16,17].

Additionally, comorbidity is an important predictor for the deterioration of health-related quality of life (HRQOL) in older patients [18,19]. HRQOL is increasingly important in clinical and decision-making processes given that this concept covers a wide range of aspects that treatment can affect, such as mobility, self-care ability, and the physical and psychological health of patients [20]. However, studies measuring the impacts of comorbidity on HRQOL in older patients suffering from falls are limited, thus, recognition of the influence and extent of comorbidities is necessary to identify appropriate interventions to improve HRQOL. Therefore, this study aims to examine comorbidity patterns and their associations with HRQOL in older patients admitted to hospital due to fall injuries in a delta province of Vietnam.

## 2. Materials and Methods

### 2.1. Design and Sample

Data of this cross-sectional study were collected from seven hospitals: One hospital at the provincial level (Thai Binh Provincial General Hospital) and six hospitals at the district level (Kien Xuong, Quynh Phu, Tien Hai, Thai Thuy, Dong Hung, and Hung Ha District General Hospitals). A convenient sampling method was applied to recruit patients who were 60 years old or older, were admitted to hospital due to fall injuries, and had normal cognition and could answer the questionnaire within 15 minutes. A total of 430 patients were invited, and 405 patients accepted to participate in the study (response rate: 94.2%).

### 2.2. Variables

After receiving written informed consents from patients, trained undergraduate medical students administered a structured questionnaire to the participants. We asked them to report the following information: Age, gender, living area (rural/urban), living arrangements (spouse/children/alone/other), caregiver (spouse/children/other), type of patient (inpatient/outpatient), history of falls (fall experienced within the past 12 months and the number of falls in the past 12 months), currently smoking (yes/no), and currently consuming alcohol (yes/no). Comorbidities were examined by physicians in these hospitals for outpatients and extracted from the medical records for inpatients. 

HRQOL of the participants was assessed by using the EuroQol-5 Dimensions-5 Levels (EQ-5D-5L). This tool consists of five dimensions, namely, mobility, self-care, usual activities, pain/discomfort, and anxiety/depression. Each dimension has five levels of response, which reveals the severity of each domain, i.e., no problem, slight problem, moderate problem, severe problem, or extreme problem [20]. Each set of five responses produces a health state, which can be converted to a health utility (EQ-5D index) using a Vietnamese cross-walk value set [21]. Participants who answered with the first option were categorized into the “no problem” group, while any other responses were categorized into the “having a problem” group. The EQ-5D-5L instrument is used widely in Vietnam [22,23,24,25,26,27]. The Vietnamese population norm of the EQ-5D index was 0.91 (SD = 0.15) [22].

### 2.3. Statistical Analysis

Stata version 15.0 (Stata Corp. LP, College Station, TX, USA) was used for the data analysis. The chi-squared test was used to identify differences in the clinical characteristics between inpatients and outpatients. Differences in the EQ-5D index among different comorbidities were examined using the Mann–Whitney test and the Kruska–Wallis test due to non-normal distribution of the EQ-5D index data. Univariate and multivariate Tobit regressions (or censored regression) were used to examine the associations between comorbidity and the EQ-5D index. The associations were adjusted for potential confounders, such as socio-demographic characteristics (age, gender, living area), living arrangements, caregivers, behaviors (smoking and alcohol drinking), history of falls, and type of patient (inpatient or outpatient).

### 2.4. Ethical Approval

This study was approved by the Institutional Review Board of Thai Binh University of Medicine and Pharmacy (Code: 764.1/HDDD).

## 3. Results

Among the 405 participants, 62.7% were outpatients and 37.3% were inpatients. The mean age was 71.9 (SD = 9.0) years old. Most patients were female (60.0%) and living in rural areas (92.1%). The majority of participants lived with spouses (58.0%) and had spouses as caregivers (52.4%). Respectively, 80.3% and 19.8% of participants were non-smokers and alcohol drinkers. The percentage of patients with comorbidities was 75.6%. Hypertension (33.1%) and osteoarthritis (33.6%) were the most common comorbidities, following by lumbar spine/cervical spine diseases (21.7%) and cardiovascular disease (12.6%). Within the last 12 months, 40.5% of participants experienced a fall, with the average number of falls being 2.0 (SD = 1.2). Among inpatients, the mean duration of hospitalization was 8.0 (SD = 3.6) days (Table 1).

**Figure 1:**
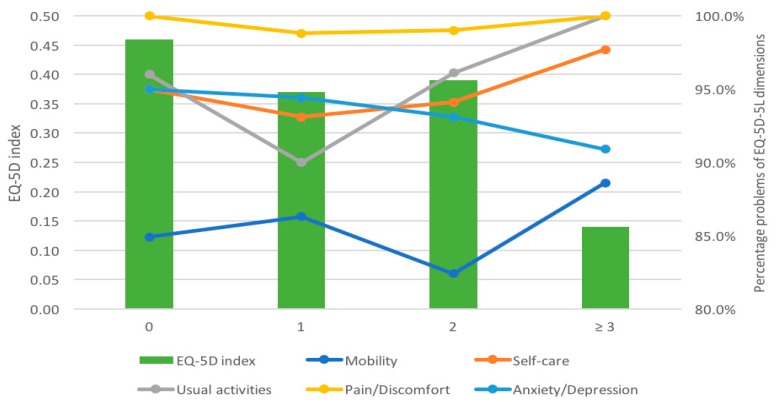
Dimensions and index of the EuroQol-5 Dimensions-5 Levels instrument according to the number of comorbidities.

Figure 1 illustrates the distribution of participants having problems in each dimension according to the number of comorbidities. Overall, more than 80% of participants reported problems in each dimension. Regarding the number of comorbidities, the proportion of participants suffering from mobility problems was the lowest (from 84.9% to 88.6%), while the rate of pain/discomfort problems was the highest (from 98.8% to 100%). A statistically significant difference was found regarding usual activity across the number of comorbidities (*p* = 0.031). Figure 1 also shows that participants without comorbidities had a mean EQ-5D index of 0.46 (SD = 0.30). The lowest EQ-5D index was 0.14 (SD = 0.40) for participants with three comorbidities or more.

**Figure 2:**
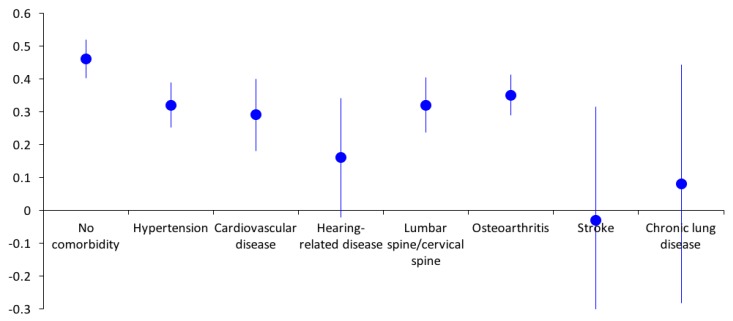
EQ-5D-5L index by chronic conditions.

As seen in Figure 2, stroke patients had the lowest mean EQ-5D index (mean = −0.03, SD = 0.43), following by chronic lung disease (mean = 0.08, SD = 0.50). Participants with hypertension had the highest mean EQ-5D index (mean = 0.39, SD = 0.36). However, all participants with comorbidities had lower EQ-5D indexes compared to those without comorbidities.

Table 2 presents the results of the univariate and multivariate regression models. In the unadjusted model, hearing-related disease, stroke, and chronic lung disease were associated with a remarkable decrease in the EQ-5D index, of which stroke had the highest effect on the HRQOL (Coefficient (Coef.) = −0.41; 95%CI = −0.71; −0.11). Having three comorbidities reduced the EQ-5D index by 0.32 (Coef. = −0.41; 95%CI = −0.45; −0.19) compared to those with no comorbidities. Participants with multiple comorbidities reported a decrease of 0.08 (Coef. = −0.08; 95%CI = −0.11; −0.04) in the EQ-5D index compared to those who did not have multiple comorbidities.

In the adjusted model, lumbar spine/cervical spine disease (Coef. = −0.10; 95%CI = −0.18; −0.03) and stroke (Coef. = −0.36; 95%CI = −0.61; −0.10) were found to be associated with significantly decreased EQ-5D indexes. Participants with three comorbidities had EQ-5D indexes that were reduced by 0.20 (Coef. = −0.20; 95%CI = −0.31; −0.09) in comparison with those without comorbidities.

## 4. Discussion

This study enriches the current literature regarding the effect of comorbidities on the HRQOL of older patients experiencing falls in Vietnam. Our results emphasize a high prevalence of comorbidities and low HRQOL in this population and a significant reduction of HRQOL among patients suffering from certain comorbidities such as lumbar spine/cervical spine diseases and stroke. The findings of this study could serve as a foundation for further interventions to improve HRQOL in older patients who experience falls in Vietnam.

The comorbidity rate in our sample was 75.6%, which is much higher than other populations in other settings such as 18.0% in Italy [28], 25.8% in Australia [8], and 45.3% in the United States [29]. These variances might be justified by the difference in study designs and settings. For example, in Italy, the sample included patients who were nursing-home residents [28]. Meanwhile, in Australia, the study population was only selected in emergency departments, which deals with acute care [8]. Our findings approximately equaled those of Korea, who reported 84.1% comorbidity [15]. Hypertension, osteoarthritis, and lumbar spine/cervical spine disease were the three most prevalent comorbidities in our sample, which was also different from previous findings. A study in Australia indicated that diabetes, renal disease, and dementia were the most common comorbidities [8]. Notably, the prevalence of chronic lung disease in this study was only 1.7%, which was lower than the prevalence of chronic obstructive pulmonary disease (COPD) among non-smokers over 40 years old in Vietnam (8.1%) [30]. Additionally, we found a discrepancy between chronic lung disease prevalence and cardiovascular prevalence in our sample. This was different from a previous study which indicated a strong association between COPD and cardiovascular illness [31]. This difference may have been be due to the under-diagnosis of COPD (e.g., limited access to pulmonary function evaluation, limited perception of the disease itself in old patients, cultural and/or economic barriers). However, the remarkably high prevalence of comorbidities in our current study raises urgent need of care planning for controlling comorbidities in older patients experiencing falls.

In our current study, even among hospitalized patients without comorbidities, the EQ-5D index (0.46) was much lower than that of the general older population (mean = 0.81) [22], suggesting that falls are responsible for a remarkable decrease in HRQOL. In our multivariate regression, only lumbar spine/cervical spine disease and stroke contributed to the reduction of HRQOL. This finding was in line with a previous study in older diabetes patients [32], but was different from dementia patients [33]. Additionally, the HRQOL of patients with one or two comorbidities might not differ from those without comorbidities, but the magnitude of reduction was substantial for those having three or more comorbidities; the adjusted EQ-5D index decreased 0.20 points in these patients compared to those with no comorbidities. This finding aligned with previous work in other populations in Vietnam [34] as well as across the world [33,35]. Unfortunately, we did not find any literature examining the influence of comorbidities on HRQOL among older fall patients; this indicates a need for other studies to validate our findings.

Several implications can be drawn from our study. First, given the negative impacts of comorbidities on HRQOL, older patients with comorbidities should be screened and monitored carefully due to their risk for impaired HRQOL. These patients have the potential to gain the highest benefits from supportive interventions. Second, further studies in the older, fall-experiencing population should collect data regarding comorbidities for adjustment, which would improve the robustness of our finding interpretations and associations. Third, routine measurement of chronic comorbidities and HRQOL using well-validated instruments in clinical settings should be recommended in order to monitor the effectiveness of treatment approaches as well as the enhancement of treatment outcomes. 

This study contained several limitations. First, we did not measure the severity and duration of each comorbidity, which might potentially influence the HRQOL of participants. Second, a cross-sectional design was used in this study; therefore, any changes in HRQOL before and after suffering from a specific comorbidity cannot be explained. Moreover, some factors such as nutritional status, body mass index, and muscle wasting were not measured [36,37], which may be potential confounding factors affecting the relationship between comorbidities and HRQOL. Therefore, additional longitudinal studies should be implemented to fill this gap. Third, a convenient sampling method restricted the generalizability of our results to other hospitals in Vietnam.

## 5. Conclusions

To conclude, this study underlined a significantly high proportion of comorbidities in older patients admitted to the hospital due to falls in Vietnam. In addition, the existence of comorbidities was associated with deteriorating HRQOL. Frequent monitoring and screening comorbidities are critical to determining which individuals are most in need of HRQOL enhancement. 

## Figures and Tables

**Table 1:** Sociodemographic and clinical characteristics of respondents.

Characteristic	n	%
**Gender**		
Male	162	40.0
Female	243	60.0
**Location**		
Urban	32	7.9
Rural	373	92.1
**Living arrangements**		
Spouse	235	58.0
Alone	17	4.2
Children	122	30.1
Other	31	7.7
**Caregiver**		
Spouse	212	52.4
Children	158	39.0
Other	35	8.6
**Smoking**	34	8.4
**Alcohol drinking**	80	19.8
**Type of patients**		
Inpatient	151	37.3
Outpatient	254	62.7
**Comorbidity**		
Hypertension	134	33.1
Cardiovascular disease	51	12.6
Hearing-related disease	16	4.0
Lumbar spine/cervical spine disease	88	21.7
Osteoarthritis	136	33.6
Stroke	6	1.5
Chronic lung disease	7	1.7
Other diseases	59	14.6
**Number of comorbidities**		
0	99	24.4
1	160	39.5
2	102	25.2
≥3	44	10.9
**Fall in the past 12 months**	164	40.5
	**Mean**	**SD**
**Age**	71.9	9.0
**Number of falls in the last 12 months**	2.0	1.2

**Table 2:** Correlations between EQ-5D-5L index and comorbidity.

Characteristics	Model 1	Model 2
	Coefficient (95%CI) ^1^	Coefficient (95%CI) ^2^
**Comorbidity**		
Hypertension	−0.06 (−0.14; 0.01)	0.01 (−0.06; 0.07)
Cardiovascular disease	−0.09 (−0.20; 0.01)	−0.06 (−0.16; 0.03)
Hearing-related disease	−0.22 (−0.41; −0.03) *	−0.07 (−0.23; 0.09)
Lumbar spine/cervical spine disease	−0.07 (−0.15; 0.02)	−0.10 (−0.18; −0.03) *
Osteoarthritis	−0.03 (−0.11; 0.05)	−0.03 (−0.10; 0.04)
Stroke	−0.41 (−0.71; −0.11) *	−0.36 (−0.61; −0.10) *
Chronic lung disease	−0.29 (−0.57; −0.01) *	−0.10 (−0.33; 0.14)
Others	−0.10 (−0.20; 0.00)	−0.08 (−0.17; 0.01)
**Number of comorbidities**		
0	ref.	ref.
1	−0.09 (−0.18; 0.0)	−0.05 (−0.12; 0.03)
2	−0.07 (−0.17; 0.03)	−0.02 (−0.11; 0.06)
3	−0.32 (−0.45; −0.19) *	−0.20 (−0.31; −0.09) *
**Multi-morbidity (≥2 diseases)**		
No	ref.	ref.
Yes	−0.08 (−0.11; −0.04) *	−0.04 (−0.08; −0.01) *

^1^ Crude coefficient; ^2^ adjusted for age, sex, living location, smoking status, alcohol use, history of falls in the last 12 months, number of falls in the last 12 months, living arrangements, caregivers, and type of patient. * *p* < 0.05.
